# Supplementary material for: Induction of cross-neutralizing antibodies by a permuted hepatitis C virus glycoprotein nanoparticle vaccine candidate
Source: Nat Commun. 2022 Nov 25;13:7271. doi: 10.1038/s41467-022-34961-8 (PMC9700739; doi:10.1038/s41467-022-34961-8)
Supplement: Supplementary file 1 — Supplementary Information [file 41467_2022_34961_MOESM1_ESM.pdf]

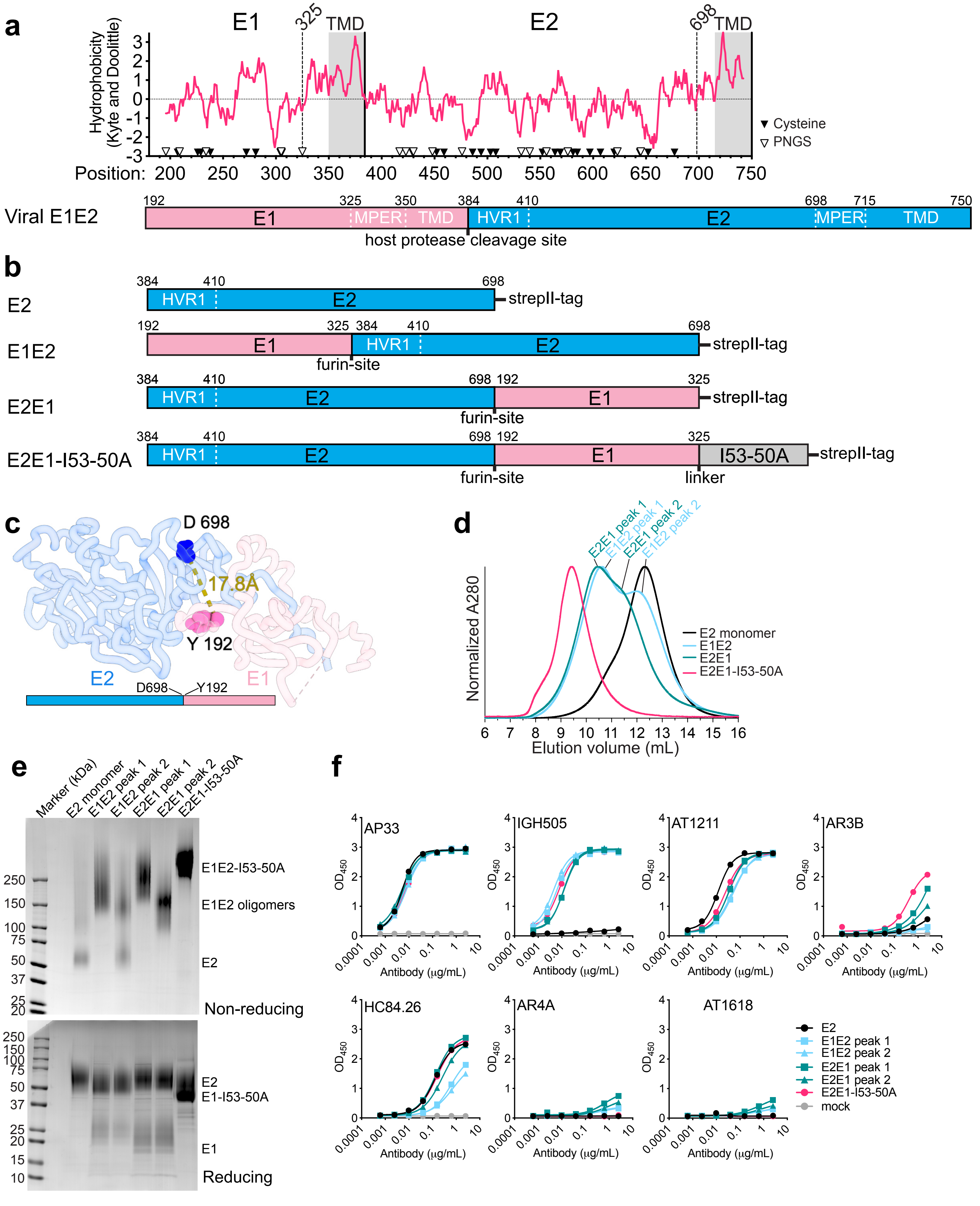

Figure S1. Design and characterization of recombinant HCV glycoproteins. **a.** Hydrophobicity plot of full-length AMS0232 E1E2. Amino acid numbering is indicated below. The transmembrane domain (TMD), E1 ectodomain truncation (325), E2 ectodomain truncation (698), PNGS and cysteines are indicated. **b.** Schematic overview of the different recombinant constructs. **c.** Structure of membrane-extracted E1E2 with the distance between D698 and Y196 of the switched C- and N-termini of E2E1 indicated (PDB: 7T6X)[1]. **d.** SEC profiles (Superdex200 column) of E2 monomer, E1E2, E2E1 and E2E1-I53-50A. **e.** Reducing and non-reducing SDS-PAGE analysis of SEC purified proteins from (c). **f.** ELISA binding curves of antibodies targeting different epitopes on E1 and E2. Source data are provided as a Source Data file.

E2

*MDAMKRGLCCVLLLCGAVFVSVTG*QTYVTGGTAARATSGLANFFSPGAKQDVQLINTNGSWHINR
TALNCNTSLETGWIAGLFYLNKFNSSGCPERMASCRPLADFAQGWGPISYANGSGPDHRPYCWHY
PPKPCGIVSAKSVCGPVYCFTPSPVVVGTTNKLGAPTYSWGENETDVFVLNNTRPPLGNWFGCTW
MNSTGFTKVCGAPPCAIGGVGNNTLHCPTDCFRKHPEATYSRCGSGPWITPRCLVDYPYRLWHYP
CTINYTRFKVRMYIGGVEHRLDAACNWTRGERCDLEDRDRSELSPLLLSTTQWQVLPCSFTTLPA
LSTGLIHLHQNIVDGSGSGGRSGWSHPQFEK

E1E2

*MDAMKRGLCCVLLLCGAVFVSVTG*YQVRNSTGLYHVTNDCPNSSIVYETADAILHTPGCVPCVRE
GNASRCWVPMTPTVATRDGKLPATQLRRHIDLLVGSATLCSALYVGDLCGSVFLVGQLFTFSPRR
HWTTQDCNCSIYPGHVTGHRMAWDMMMNGGSGGSGGSGGSGGSRRRRRRQTYVTGGTAARATSGL
ANFFSPGAKQDVQLINTNGSWHINRTALNCNTSLETGWIAGLFYLNKFNSSGCPERMASCRPLAD
FAQGWGPISYANGSGPDHRPYCWHYPPKPCGIVSAKSVCGPVYCFTPSPVVVGTTNKLGAPTYSW
GENETDVFVLNNTRPPLGNWFGCTWMNSTGFTKVCGAPPCAIGGVGNNTLHCPTDCFRKHPEATY
SRCGSGPWITPRCLVDYPYRLWHYPCTINYTRFKVRMYIGGVEHRLDAACNWTRGERCDLEDRDR
SELSPLLLSTTQWQVLPCSFTTLPALSTGLIHLHQNIVDGSGSGGRSGWSHPQFEK

E2E1

*MDAMKRGLCCVLLLCGAVFVSVTG*QTYVTGGTAARATSGLANFFSPGAKQDVQLINTNGSWHINR
TALNCNTSLETGWIAGLFYLNKFNSSGCPERMASCRPLADFAQGWGPISYANGSGPDHRPYCWHY
PPKPCGIVSAKSVCGPVYCFTPSPVVVGTTNKLGAPTYSWGENETDVFVLNNTRPPLGNWFGCTW
MNSTGFTKVCGAPPCAIGGVGNNTLHCPTDCFRKHPEATYSRCGSGPWITPRCLVDYPYRLWHYP
CTINYTRFKVRMYIGGVEHRLDAACNWTRGERCDLEDRDRSELSPLLLSTTQWQVLPCSFTTLPA
LSTGLIHLHQNIVDGGSGGSGGSGGSGGSRRRRRRYQVRNSTGLYHVTNDCPNSSIVYETADAIL
HTPGCVPCVREGNASRCWVPMTPTVATRDGKLPATQLRRHIDLLVGSATLCSALYVGDLCGSVFL
VGQLFTFSPRRHWTTQDCNCSIYPGHVTGHRMAWDMMMNGSGSGGRSGWSHPQFEK

E2E1-I53-50A

*MDAMKRGLCCVLLLCGAVFVSVTG*QTYVTGGTAARATSGLANFFSPGAKQDVQLINTNGSWHINR
TALNCNTSLETGWIAGLFYLNKFNSSGCPERMASCRPLADFAQGWGPISYANGSGPDHRPYCWHY
PPKPCGIVSAKSVCGPVYCFTPSPVVVGTTNKLGAPTYSWGENETDVFVLNNTRPPLGNWFGCTW
MNSTGFTKVCGAPPCAIGGVGNNTLHCPTDCFRKHPEATYSRCGSGPWITPRCLVDYPYRLWHYP
CTINYTRFKVRMYIGGVEHRLDAACNWTRGERCDLEDRDRSELSPLLLSTTQWQVLPCSFTTLPA
LSTGLIHLHQNIVDGGSGGSGGSGGSGGSRRRRRRYQVRNSTGLYHVTNDCPNSSIVYETADAIL
HTPGCVPCVREGNASRCWVPMTPTVATRDGKLPATQLRRHIDLLVGSATLCSALYVGDLCGSVFL
VGQLFTFSPRRHWTTQDCNCSIYPGHVTGHRMAWDMMMNGSGGSGGSGGSGGSEKAAKAEEAARK
MEELFKKHKIVAVLRANSVEEAIEKAVAVFAGGVHLIEITFTVPDADTVIKALSVLKEKGAIIGA
GTVTSVEQCRKAVESGAEFIVSPHLDEEISQFCKEKGVFYMPGVMTPTELVKAMKLGHDILKLFP
GEVVGPEFVKAMKGPFPNVKFVPTGGVDLDNVCEWFDAGVLAVGVGDALVEGDPDEVREKAKEFV
EKIRGCTEGSLEWSHPQFEK

Signal peptide
E1
E2
I53-50A
strepII-tag

Figure S2. Amino acid sequences of the HCV glycoproteins. All constructs listed here are based on the AMS0232 sequence (with I442F mutation).

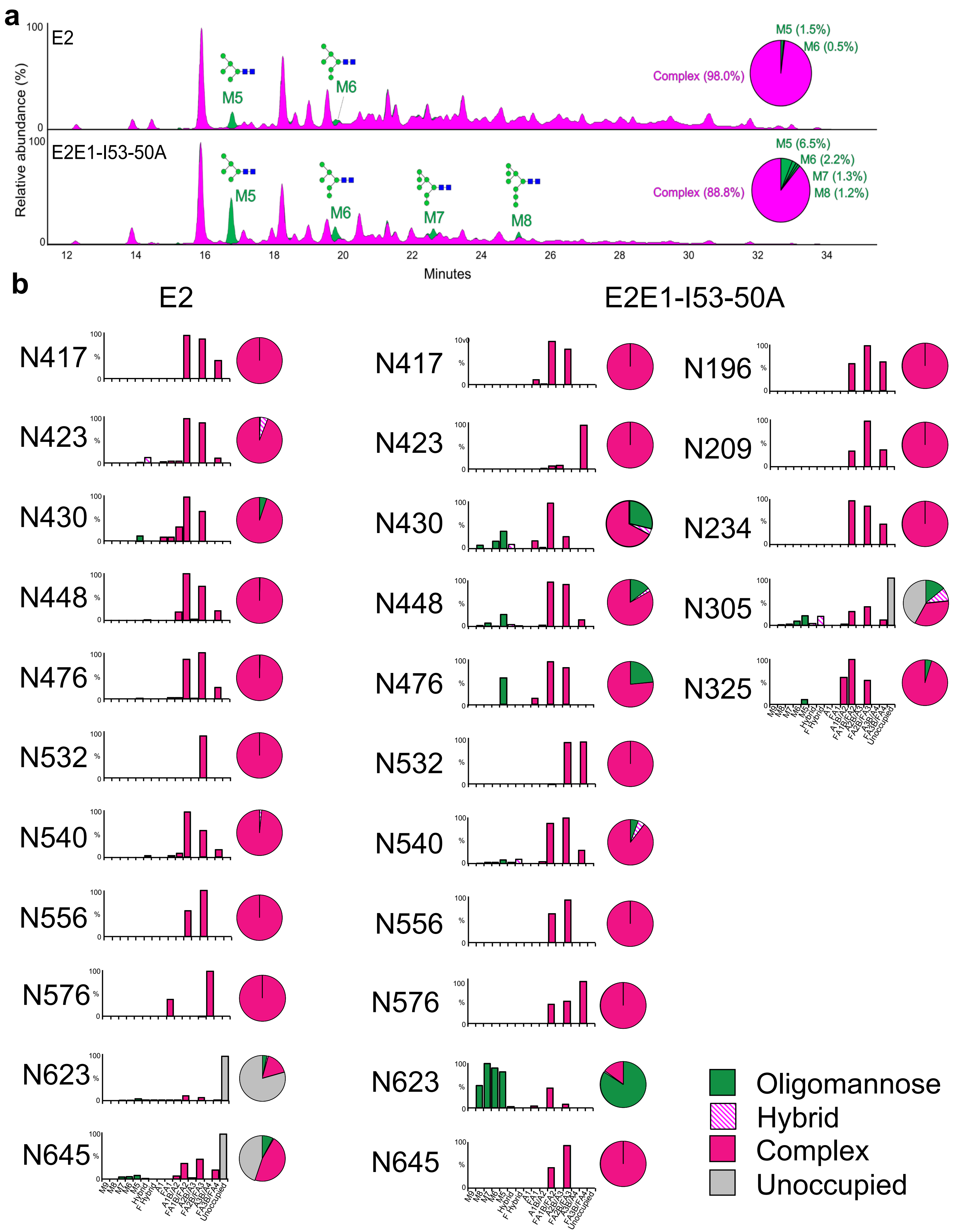

Figure S3. Glycosylation of E2 and E2E1-I53-50A. a. Overall glycan profile of AMS0232 E2 and E2E1-I53-50A as determined by hydrophilic interaction liquid chromatography-ultra performance liquid chromatography. **b.** Detailed site-specific glycosylation analysis of all PNGS in E2 monomer and E2E1-I53-50A trimer. Bar graphs indicate relative abundance of the different glycan species depicted on the x-axis: oligomannose species are indicated by M5-9 ($Man_{5\text{-}9}GlcNAc_2$). For the complex glycans, An indicates the number (n) of antennae (e.g. A3 = triantennary); F indicates the presence of a core fucose. More detailed description of the glycan abbreviations can be found in Behrens et al.[2]. Circle diagram contains the aggregated percentage of complex (pink), oligomannose (green) or hybrid (shaded pink/white) glycans or the percentage of unoccupied (grey) (also depicted in Figure 1c).

Figure S4

a

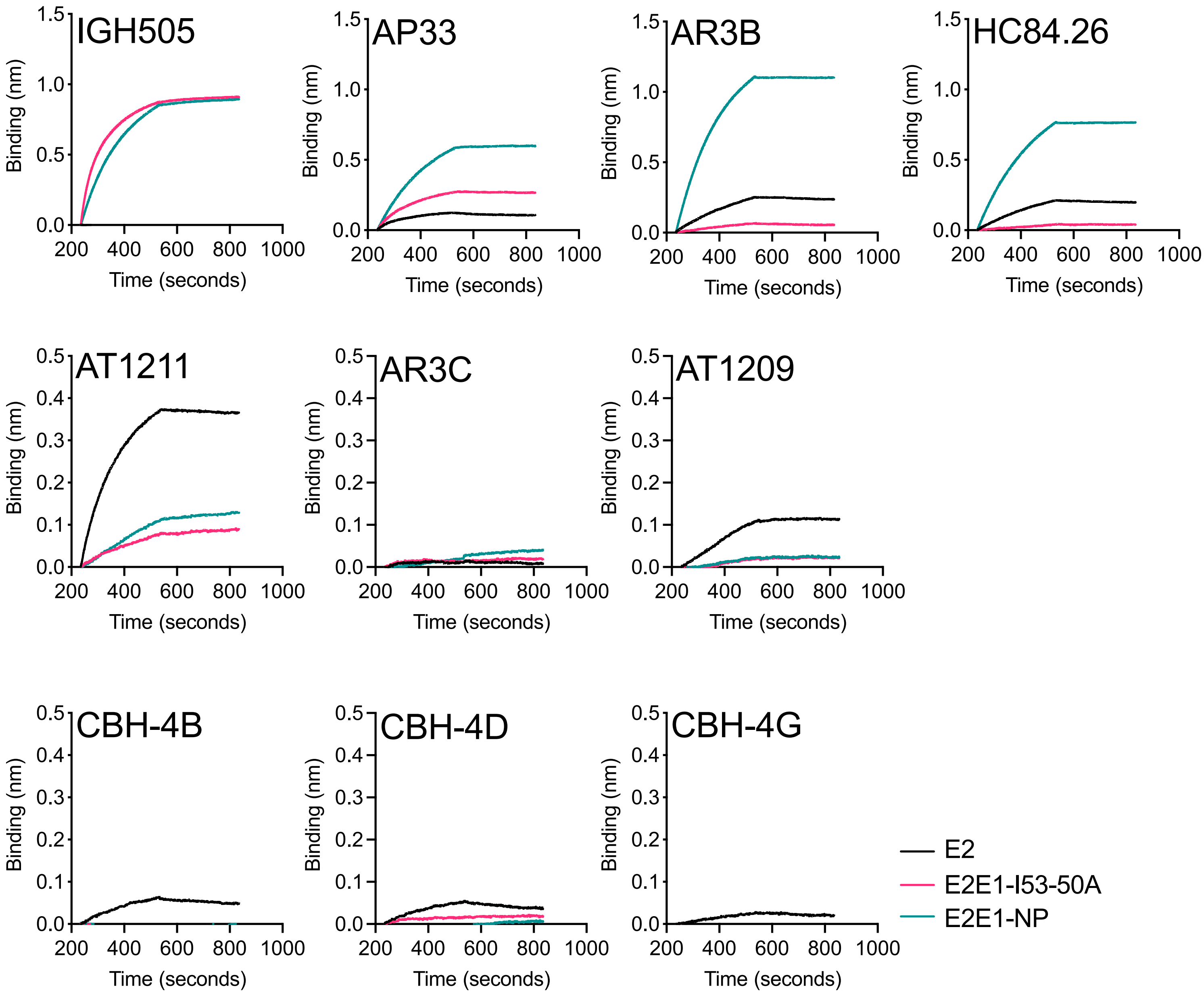

b

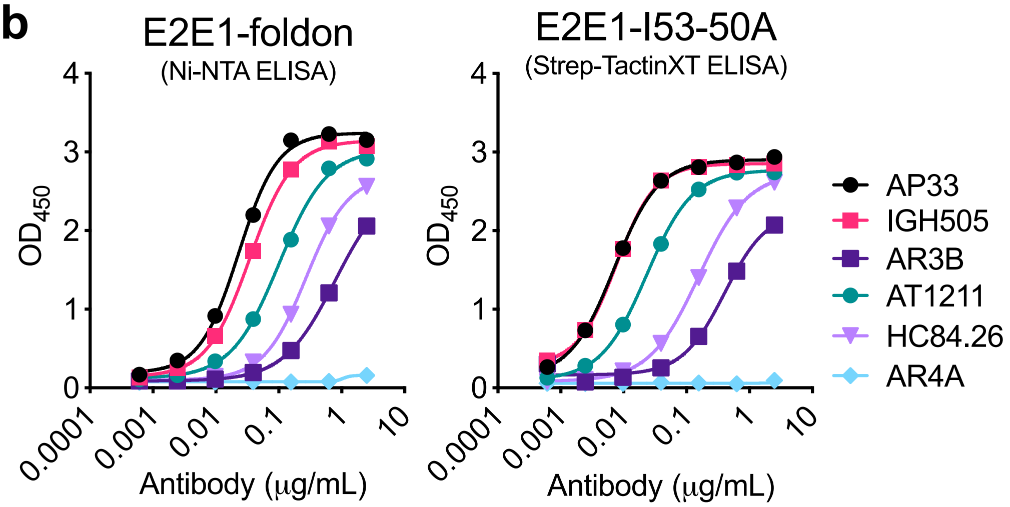

Figure S4. Antigenicity of recombinant HCV glycoproteins. a. BLI measurements of AMS0232 E2, E2E1-I53-50A and E2E1-NP (100 nM E2) binding to mAbs that were first immobilized on protein A sensors. Related to Figure 1h. Representative curves from one of two independent experiments. **b.** Antibody binding measured by ELISA of His-tagged E2E1-foldon captured on Ni-NTA plates compared to StrepII-tagged E2E1-I53-50A captured on Strep-TactinXT plates (from Figure S1f). Source data are provided as a Source Data file.

Figure S5

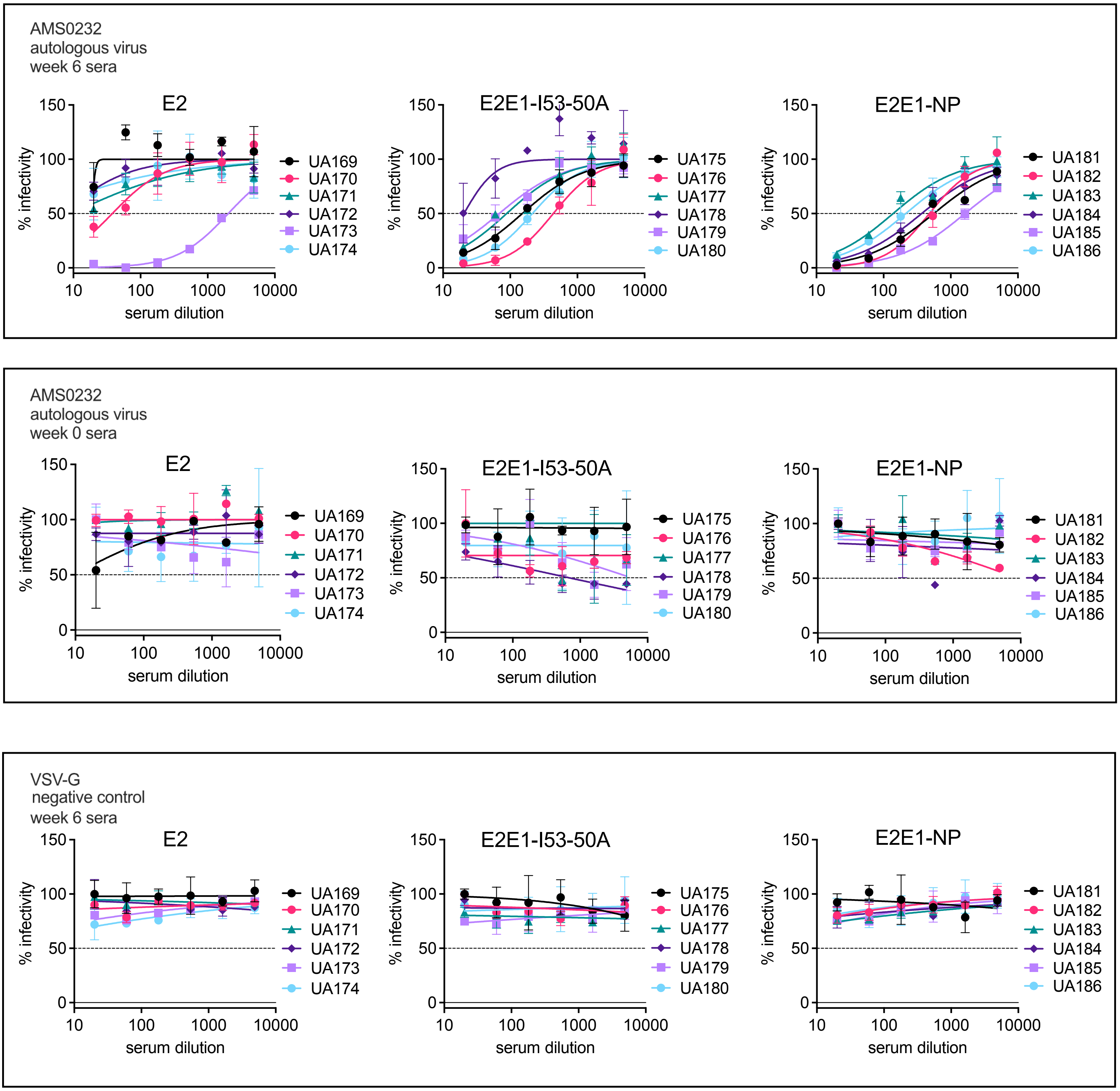

Figure S5. Neutralization of AMS0232 HCVpp and VSV-G by rabbit sera. Neutralization of sera from rabbits immunized with E2 (left), E2E1-I53-50A (middle) or E2E1-NP (right). Depicted are the neutralization curves of the post-boost sera (week 6) against AMS0232 HCVpp (top), pre-immunize sera (week 0) against AMS0232 HCVpp and post-boost sera (week 6) against VSV-G (negative control virus). Depicted are the mean values and S.D. of a single experiment performed in duplo. Source data are provided as a Source Data file.

Figure S6

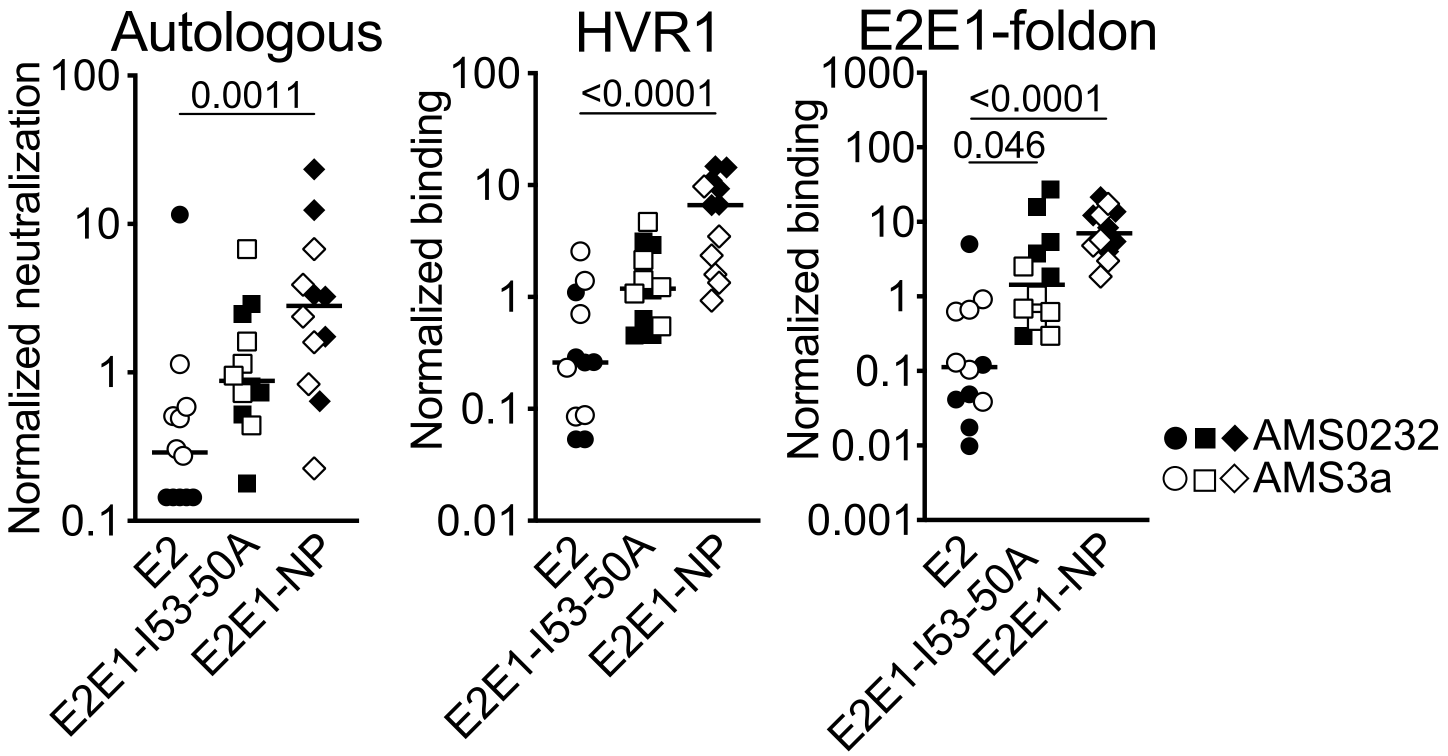

Figure S6. Normalized autologous neutralizing and binding antibody responses. Collated immunogenicity data of the AMS0232 and AMS3a-based vaccination studies. The endpoint binding titers (Figure 2b, d and Figure 3d, e) and autologous neutralization ID50 titers (Figure 2e and Figure 3g) were normalized against the geometric mean titer (based on n=18 titers for each immunogenicity study). The geometric mean titer was set to 1.0. Horizontal lines indicate the median values. Groups were compared using a Kruskal-Wallis test followed by Dunn's post-hoc test. *n* = 12 rabbit sera per group. Source data are provided as a Source Data file.

Figure S7

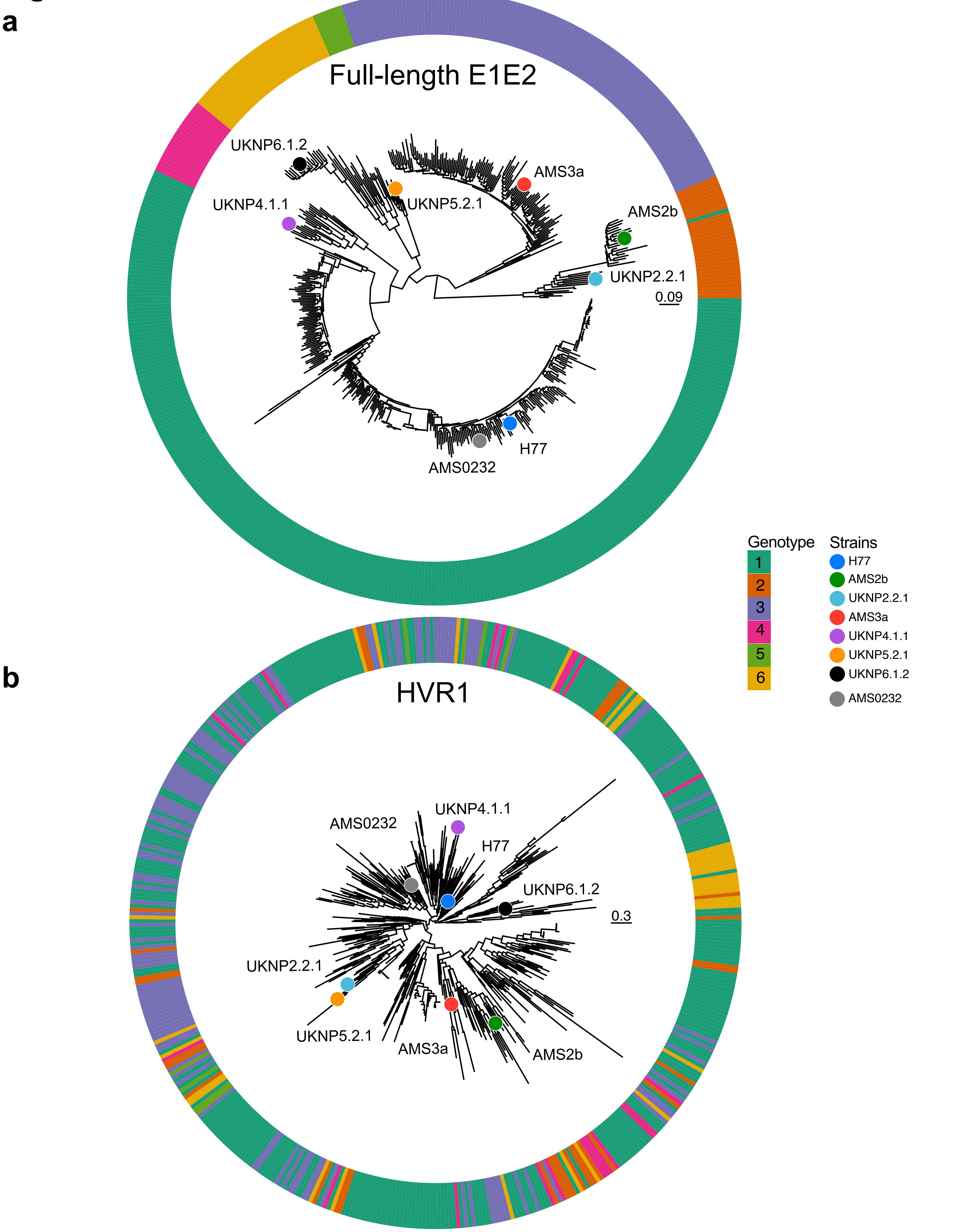

Figure S7. Amino acid diversity of E1E2 and HVR1. a. Maximum likelihood tree of 512 randomly selected E1E2 sequences from the HCV-GLUE database[3]. **b**. Maximum likelihood tree of the HVR1 of the same 512 isolates in (a). Note that full-length E1E2 sequences cluster by genotype, but the HVR1 sequences do not. Amino-acid sequences were aligned using MAFFT and manually checked for correct alignment[4]. ML-Trees were inferred using IQ-TREE with a GTR+Gamma+Invariant Sites substitution model[5].

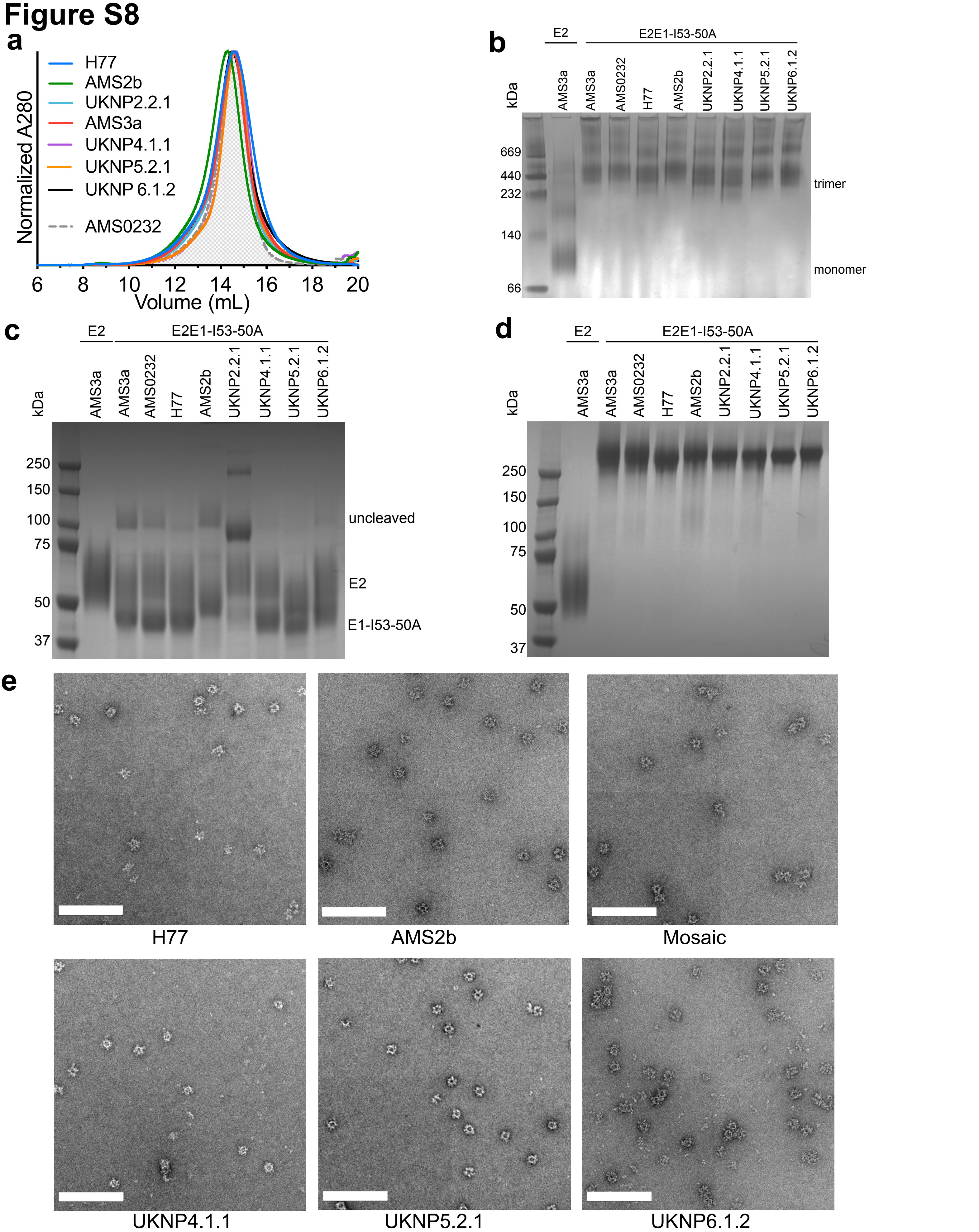

Figure S8. Characterization of E2E1-I53-50A trimers based on different primary HCV isolates. a. SEC profiles of Strep-TactinXT-purified E2E1-I53-50A. AMS0232 E2E1-I53-50A trimer chromatogram is shown in grey for comparison. **b**. BN-PAGE analysis of SEC-purified E2E1-I53-50A trimers. E2 monomer is shown for comparison. From a single experiment. **c**. Reducing SDS-PAGE of SEC-purified E2E1-I53-50A trimers. Uncleaved E2E1-I53-50A is indicated. From a single experiment. **d**. Non-reducing SDS-PAGE of SEC-purified E2E1-I53-50A trimers. From a single experiment. **e**. Representative raw NS-EM images of E2E1-NPs assembled from E2E1-I53-50A trimers. Related to Figure 4c. The white scale bar represents 200 nm. Source data for *a* are provided as a Source Data file and at the end of the Supplemental Figure file (b-d).

Figure S9

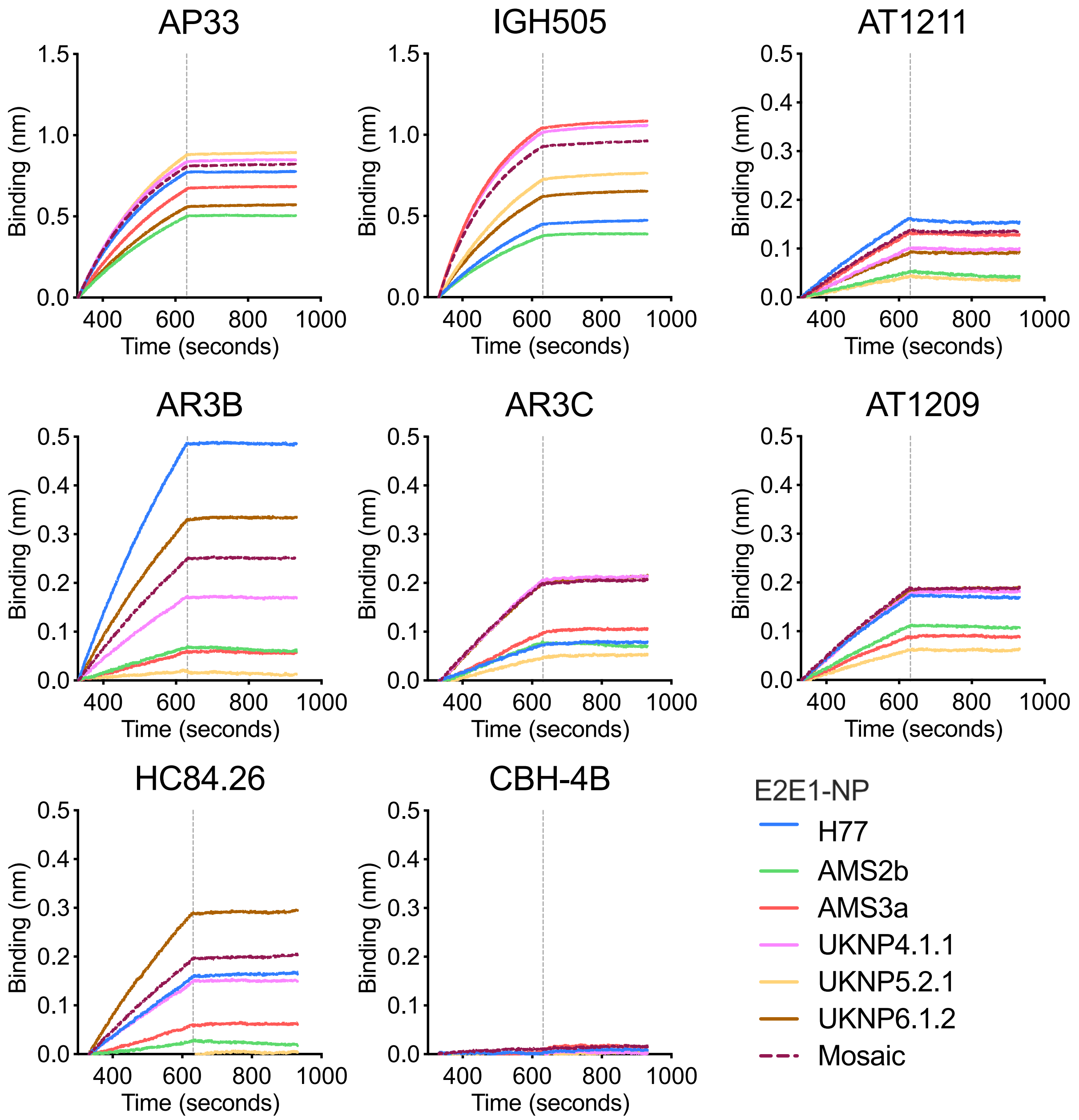

Figure S9. Antigenicity of E2E1-NPs. BLI measurements of E2E1-NPs (100 nM of E2) binding to mAbs that were first immobilized on protein A sensors. Related to Figure 4d. Source data are provided as a Source Data file.

Figure S10

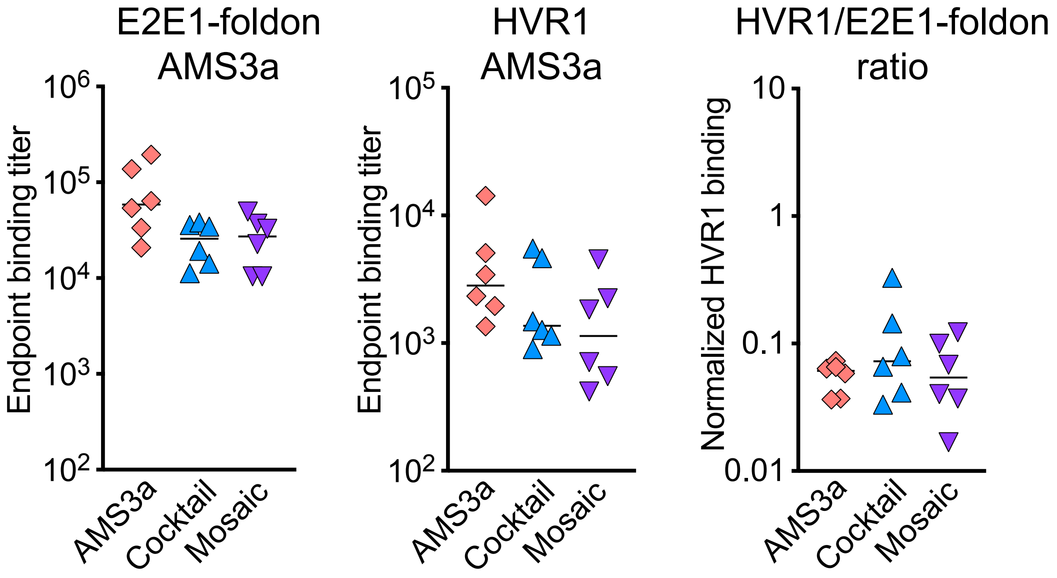

Figure S10. Binding of week 6 sera from rabbits immunized with AMS3a E2E1-NPs, cocktail of E2E1-NPs or mosaic E2E1-NPs. Endpoint binding titers were measured against AMS3a E2E1-foldon (left), AMS3a HVR1 (middle) and their binding ratios (right). Horizontal lines indicate the median values. Groups were compared using a Kruskal-Wallis test followed by Dunn's post-hoc test: no significant differences between groups. *n* = 6 rabbit sera per group. Source data are provided as a Source Data file.

Figure S11

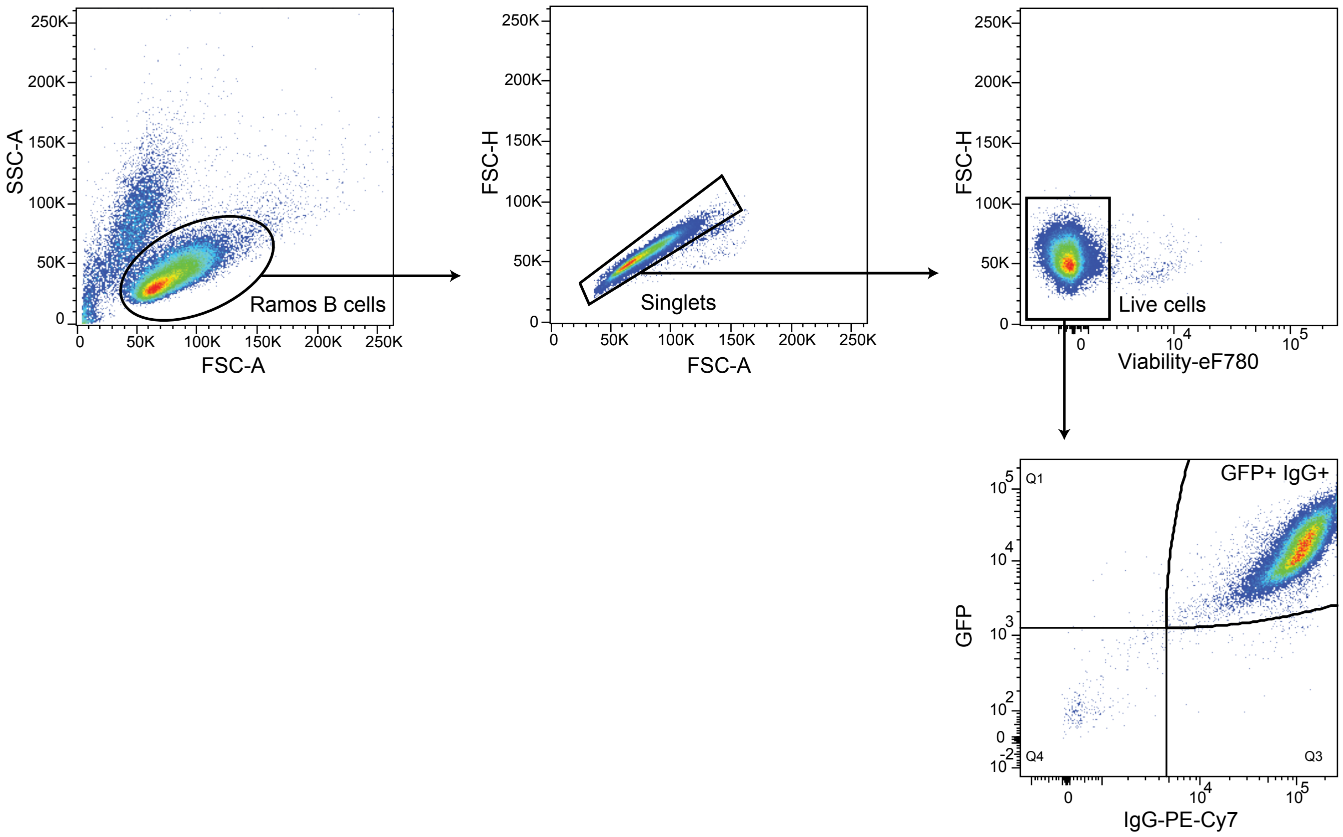

Figure S11. Gating stratey to identify B cells expressing the transduced AR3C B cell receptor (GFP+/IgG+). Transduced live Ramos B cells were sorted based double positive staining for GFP and IgG expression (lower right panel).

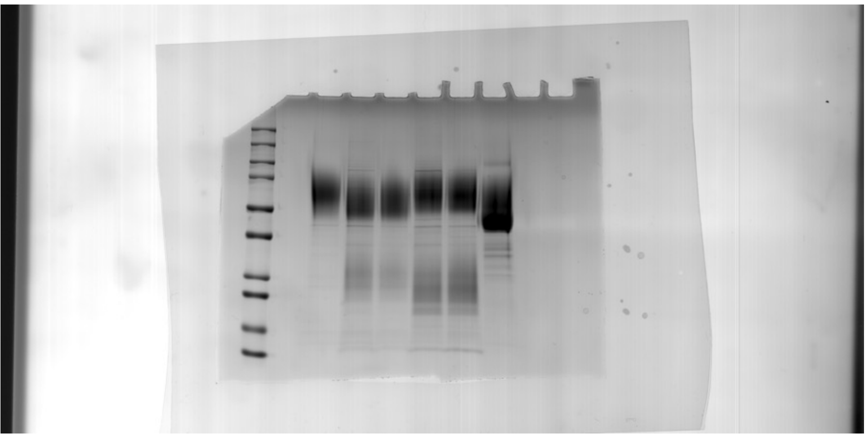

Figure S1e uncropped

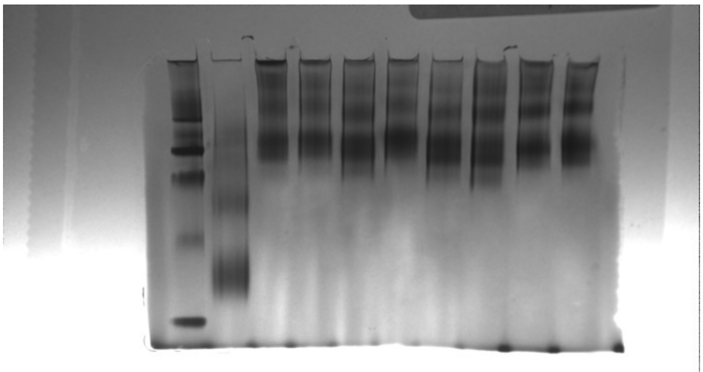

Figure S8b uncropped

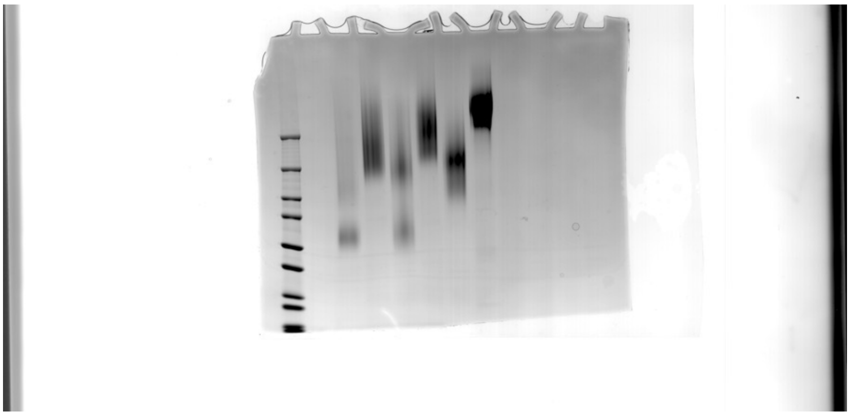

Figure S1e uncropped

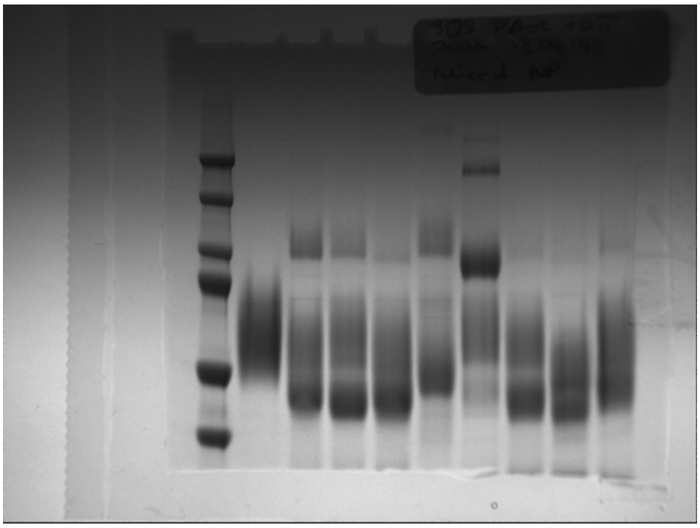

Figure S8c uncropped

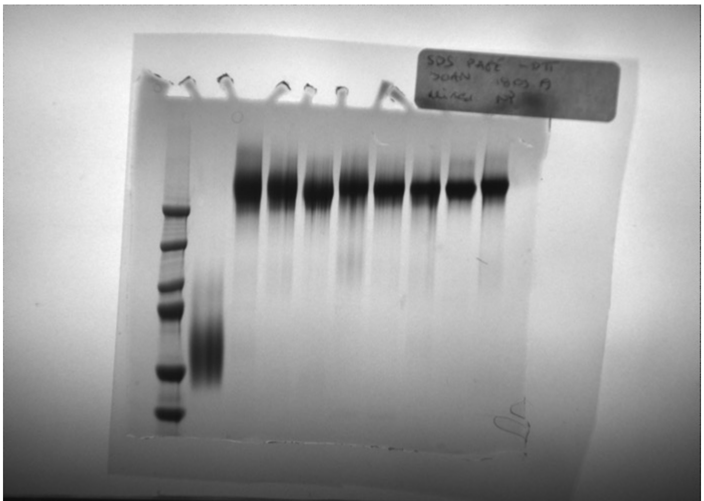

Figure S8d uncropped

Figure S12. Uncropped versions of SDS PAGE gels and BN PAGE gels from the supplemental figures

Table S1. Midpoint neutralization (ID_{50}) titers for sera from rabbits immunized with different recombinant HCV glycoprotein antigens.

Sera from week 6 (two weeks post-boost) were tested against a panel of HCV pseudoparticles in a standard neutralization assay. Upper table: neutralizing titers for sera from rabbits immunized with AMS0232-based E2, E2E1-I53-50A and E2E1-NP (related to Figure 2). Immunizations were carried out by Covance (Denver, USA). Bottom table: neutralizing titers for sera from rabbits immunized with AMS3a-based E2, E2E1-I53-50A, E2E1-NP or a cocktail of E2E1-NPs or mosaic E2E1-NPs (related to Figure 3 and 5). Immunizations were carried out by Pocono Rabbit Farm & Laboratory (Canadensis, USA).
The boxes are colored according to their magnitude: ID_{50}: <20 in white; ID_{50}: 20-39 in light yellow; ID_{50}: 40-99 in yellow; ID_{50}: 100-299 in orange; ID_{50}: 300-999 in red; ID_{50}: >1000 in purple.

			non-matched							
	Animal ID	AMS0232 1a	H77 1a	AMS0229 1a	AMS0231 1a	AMS1b.2 1b	UKNP2.1.1 2	AMS3a.26 3	UKNP5.2.1 5	VSV-G (-) (Virus Genotype)
E2 AMS0232	UA169	<20	<20	<20	<20	<20	<20	20	<20	<20
	UA170	29	<20	<20	<20	<20	<20	22	<20	<20
	UA171	<20	<20	<20	<20	<20	<20	<20	<20	<20
	UA172	<20	<20	<20	<20	<20	<20	38	<20	<20
	UA173	1612	<20	<20	<20	273	<20	74	<20	<20
	UA174	<20	<20	<20	<20	<20	<20	22	30	<20
E2E1-I53-50A AMS0232	UA175	112	<20	24	<20	20	<20	29	397	<20
	UA176	343	<20	<20	<20	<20	<20	<20	20	<20
	UA177	72	<20	<20	<20	<20	<20	<20	<20	<20
	UA178	25	<20	<20	<20	<20	<20	<20	<20	<20
	UA179	102	<20	<20	<20	<20	<20	<20	46	<20
	UA180	402	<20	31	106	32	<20	42	103	<20
E2E1-NP AMS0232	UA181	450	92	942	184	58	24	55	40	<20
	UA182	464	<20	<20	218	183	<20	<20	527	<20
	UA183	89	<20	<20	27	<20	<20	25	40	<20
	UA184	1726	<20	<20	34	<20	<20	<20	24	<20
	UA185	3245	<20	<20	37	<20	<20	<20	205	<20
	UA186	243	<20	<20	29	33	<20	35	408	<20

		vaccine-matched (cocktail/mosaic)						non-matched											Global geometric mean titer (GGMT)		
	Animal ID	AMS3a.26 3	H77 1a	AMS2b.21 2	UKNP4.1.1 4	UKNP5.2.1 5	UKNP6.1.2 6	AMS0229 1a	AMS0230 1a	AMS0232 1a	AMS1b.2 1b	JFH1 2	UKNP2.2.1 2	UKNP2.4.1 2	UKNP3.1.2 3	UKNP3.2.2 3	AMS4d.9 4	VSV-G (-) (Virus Genotype)	Vaccine-matched	Non-matched	All
E2 AMS3a	36709	52	27	<20	<20	26	36	<20	35	<20	29	<20	<20	63	<20	<20	<20	<20	28	25	26
	36710	50	<20	<20	<20	<20	<20	<20	22	129	<20	<20	<20	<20	29	<20	37	<20	23	27	25
	36711	60	65	31	<20	<20	<20	<20	27	54	28	<20	<20	67	71	<20	<20	<20	31	30	31
	36712	31	<20	<20	<20	<20	<20	<20	40	36	<20	<20	<20	<20	69	<20	26	<20	22	26	24
	36713	116	34	<20	<20	<20	30	<20	25	37	<20	<20	23	38	<20	29	31	<20	31	26	28
	36714	28	20	<20	<20	21	45	<20	41	167	<20	<20	<20	<20	25	<20	55	<20	24	30	28
E2E1-I53-50A AMS3a	36715	74	23	<20	<20	<20	22	<20	53	130	<20	<20	41	24	67	28	54	<20	26	37	33
	36716	117	42	36	<20	30	<20	<20	21	37	<20	<20	<20	27	<20	57	<20	<20	36	24	28
	36717	97	42	<20	<20	<20	<20	<20	<20	32	<20	<20	<20	25	52	39	<20	<20	29	25	27
	36718	45	<20	<20	<20	<20	<20	<20	49	104	36	<20	21	<20	30	<20	48	<20	23	31	28
	36719	692	30	24	<20	78	44	22	76	44	<20	31	43	36	33	<20	32	<20	57	33	40
	36720	165	25	<20	<20	192	27	<20	<20	<20	24	<20	<20	22	<20	<20	<20	21	45	21	28
E2E1-NP AMS3a	36721	398	267	144	28	36	36	24	131	87	30	29	57	75	42	31	75	<20	91	50	63
	36722	163	25	<20	<20	94	65	<20	53	179	<20	25	<20	23	34	<20	48	<20	46	33	37
	36723	85	<20	<20	<20	46	<20	<20	22	45	<20	<20	23	30	<20	<20	25	<20	29	24	26
	36724	690	37	32	23	216	40	<20	63	93	140	23	31	30	26	<20	35	<20	74	38	49
	36725	242	<20	<20	<20	<20	26	<20	<20	61	<20	<20	<20	206	26	<20	<20	<20	32	29	30
	36726	23	<20	<20	24	657	22	<20	57	130	<20	61	31	<20	<20	<20	73	<20	38	36	37
Cocktail E2E1-NP H77, AMS2b, AMS3a, UKNP4.1.1, UKNP5.2.1 UKNP6.1.2	36727	100	56	690	91	2780	861	42	31	380	281	38	30	54	132	33	50	<20	308	67	119
	36728	72	101	198	28	4860	333	<20	<20	119	118	32	<20	29	24	38	22	<20	201	34	66
	36729	123	66	20	39	4860	101	<20	28	101	<20	36	34	<20	31	<20	65	<20	121	32	53
	36730	284	238	360	244	4050	447	<20	123	96	297	115	35	32	45	24	<20	<20	470	54	121
	36731	66	96	297	262	4860	159	44	45	197	35	39	51	<20	43	<20	144	<20	269	48	92
	36732	100	137	154	20	4597	1813	23	28	111	717	43	25	34	194	<20	<20	<20	266	52	96
Mosaic E2E1-NP H77, AMS2b, AMS3a, UKNP4.1.1, UKNP5.2.1 UKNP6.1.2	36733	45	661	137	40	3427	163	154	50	255	915	124	35	83	265	36	<20	<20	212	102	134
	36734	65	37	59	111	1024	205	45	118	463	43	67	60	35	55	23	157	<20	122	70	86
	36735	159	48	183	44	731	70	60	94	114	46	46	22	38	49	<20	134	<20	121	52	72
	36736	397	632	779	523	4860	66	48	69	259	724	61	27	126	121	77	56	25	566	96	187
	36737	103	192	57	80	4852	54	134	86	372	58	114	32	56	229	30	218	<20	169	97	120
	36738	34	44	36	<20	464	109	<20	42	117	56	26	28	52	25	21	36	<20	62	36	44

Supplemental references

1. Torrents de la Peña, A. et al. Structure of the hepatitis C virus E1E2 glycoprotein complex. *Science* **378**, 263–269 (2022).

2. Behrens, A.-J. et al. Composition and antigenic effects of individual glycan sites of a trimeric HIV-1 envelope glycoprotein. *Cell Rep*. **14**, 2695–706 (2016).

3. Singer, J.B. et al. GLUE: a flexible software system for virus sequence data. *BMC Bioinformatics* **19**, 1–18. (2018)

4. Katoh, K., and Standley, D.M. MAFFT Multiple Sequence Alignment Software Version 7: Improvements in Performance and Usability. *Mol. Biol. Evol*. **30**, 772–780 (2013).

5. Nguyen, L.-T. et al. IQ-TREE: A Fast and Effective Stochastic Algorithm for Estimating Maximum-Likelihood Phylogenies. *Mol. Biol. Evol*. **32**, 268–274. (2015).
