# Supplementary material for: Induction of cross-neutralizing antibodies by a permuted hepatitis C virus glycoprotein nanoparticle vaccine candidate
Source: Nat Commun. 2022 Nov 25;13:7271. doi: 10.1038/s41467-022-34961-8 (PMC9700739; doi:10.1038/s41467-022-34961-8)
Supplement: Supplementary file 3 — Reporting Summary [file 41467_2022_34961_MOESM3_ESM.pdf]

nature portfolio

Corresponding author(s): Kwinten Sliepen
Rogier W. Sanders

Last updated by author(s): Nov 11, 2022

Reporting Summary

Nature Portfolio wishes to improve the reproducibility of the work that we publish. This form provides structure for consistency and transparency in reporting. For further information on Nature Portfolio policies, see our Editorial Policies and the Editorial Policy Checklist.

Statistics

For all statistical analyses, confirm that the following items are present in the figure legend, table legend, main text, or Methods section.

n/a	Confirmed	
☐	☒	The exact sample size (*n*) for each experimental group/condition, given as a discrete number and unit of measurement
☐	☒	A statement on whether measurements were taken from distinct samples or whether the same sample was measured repeatedly
☐	☒	The statistical test(s) used AND whether they are one- or two-sided<br>*Only common tests should be described solely by name; describe more complex techniques in the Methods section.*
☒	☐	A description of all covariates tested
☐	☒	A description of any assumptions or corrections, such as tests of normality and adjustment for multiple comparisons
☐	☒	A full description of the statistical parameters including central tendency (e.g. means) or other basic estimates (e.g. regression coefficient) AND variation (e.g. standard deviation) or associated estimates of uncertainty (e.g. confidence intervals)
☐	☒	For null hypothesis testing, the test statistic (e.g. *F*, *t*, *r*) with confidence intervals, effect sizes, degrees of freedom and *P* value noted<br>*Give P values as exact values whenever suitable.*
☒	☐	For Bayesian analysis, information on the choice of priors and Markov chain Monte Carlo settings
☒	☐	For hierarchical and complex designs, identification of the appropriate level for tests and full reporting of outcomes
☐	☒	Estimates of effect sizes (e.g. Cohen's *d*, Pearson's *r*), indicating how they were calculated

Our web collection on statistics for biologists contains articles on many of the points above.

Software and code

Policy information about availability of computer code

Data collection	Microsoft Exce (v16.5)l for database management.
Data analysis	Data were analyzed using Microsoft Excel and Graphpad Prism 8.3. Statistical analyses were done using Graphpad Prism 8.3.

For manuscripts utilizing custom algorithms or software that are central to the research but not yet described in published literature, software must be made available to editors and reviewers. We strongly encourage code deposition in a community repository (e.g. GitHub). See the Nature Portfolio guidelines for submitting code & software for further information.

Data

Policy information about availability of data

All manuscripts must include a data availability statement. This statement should provide the following information, where applicable:
- Accession codes, unique identifiers, or web links for publicly available datasets
- A description of any restrictions on data availability
- For clinical datasets or third party data, please ensure that the statement adheres to our policy

Source data file is provided with this paper . Other data that support the findings of this study are available from the corresponding authors (K.S. and R.W.S.) upon reasonable request. Genbank accession codes are indicated in the Methods section and in the data availability section.

Human research participants

Policy information about studies involving human research participants and Sex and Gender in Research.

Reporting on sex and gender	*N.A*
Population characteristics	*N/A*
Recruitment	N/A
Ethics oversight	*Identify the organization(s) that approved the study protocol.*

Note that full information on the approval of the study protocol must also be provided in the manuscript.

Field-specific reporting

Please select the one below that is the best fit for your research. If you are not sure, read the appropriate sections before making your selection.

☒ Life sciences ☐ Behavioural & social sciences ☐ Ecological, evolutionary & environmental sciences

For a reference copy of the document with all sections, see nature.com/documents/nr-reporting-summary-flat.pdf

Life sciences study design

All studies must disclose on these points even when the disclosure is negative.

Sample size	No sample-size calculations were performed. >5 animals were sufficient to detect effect previously (Sliepen Nat Comm 2019).
Data exclusions	No data was excluded from analyses, except for a few outliers in the data underlying Fig 3f. (see source data for Fig. 3f).
Replication	Experiments were repeated and/or performed duplicate or triplicate. Repeats showed similar results.
Randomization	Rabbits were randomly distributed among the groups.
Blinding	Experiments were analyzed by unblinded investigators due for pragmatic reasons.

Reporting for specific materials, systems and methods

We require information from authors about some types of materials, experimental systems and methods used in many studies. Here, indicate whether each material, system or method listed is relevant to your study. If you are not sure if a list item applies to your research, read the appropriate section before selecting a response.

Materials & experimental systems

n/a	Involved in the study	
☐	☒	Antibodies
☐	☒	Eukaryotic cell lines
☒	☐	Palaeontology and archaeology
☐	☒	Animals and other organisms
☒	☐	Clinical data
☒	☐	Dual use research of concern

Methods

n/a	Involved in the study	
☒	☐	ChIP-seq
☐	☒	Flow cytometry
☒	☐	MRI-based neuroimaging

Antibodies

Antibodies used	AR4A, AT1209, AT1211, AR3C, AP33, AR3B, HC84.26 were produced in the lab. CBH-4B, 4D, 4G were provided by Steven Foung (Stanford University, USA). All other antibodies were produced using transient transfection using plasmids expressing the antibody heavy and light chains. Goat-anti-human IgG Jackson Immunoresearch (cat# 109-035-170)
Validation	AR4A, AT1209, AT1211, AR3C, AP33, AR3B, HC84.26 antibody plasmids were validatedd by sequencing.

Eukaryotic cell lines

Policy information about cell lines and Sex and Gender in Research

Cell line source(s)	HEK293 cell lines were obtained from Thermo Fisher or ATCC as described in the methods.<br>Ramos B cells were obtained from dr. Li Wu and dr. Vineet N. KewalRamani via the NIH AIDS Reagent Program
Authentication	None of the cell lines were validated.
Mycoplasma contamination	All cell lines were tested negative for mycoplasma
Commonly misidentified lines (See ICLAC register)	No commonly misidentiefied cell lines were used

Animals and other research organisms

Policy information about studies involving animals; ARRIVE guidelines recommended for reporting animal research, and Sex and Gender in Research

Laboratory animals	New Zealand White rabbits, female, start age/weight; 2.5-3kg
Wild animals	No wild animals were used in this study
Reporting on sex	All rabbits were female
Field-collected samples	No field ollected samples were used in this study.
Ethics oversight	All immunization procedures complied with all relevant ethical regulations and protocols of the Covance Institutional Animal Care and Use Committee (IACUC, study C0096-19) and the Pocono Institutional Animal Care and Use Committee (IACUC).

Note that full information on the approval of the study protocol must also be provided in the manuscript.

Flow Cytometry

Plots

Confirm that:

- [x] The axis labels state the marker and fluorochrome used (e.g. CD4-FITC).
- [x] The axis scales are clearly visible. Include numbers along axes only for bottom left plot of group (a 'group' is an analysis of identical markers).
- [x] All plots are contour plots with outliers or pseudocolor plots.
- [x] A numerical value for number of cells or percentage (with statistics) is provided.

Methodology

Sample preparation	IgM-negative Ramos B cell lines were previously transfuced with AR3C as described in the methods. Cells were then sorted for GFP+ IgG + IgM-. Cells were frozen utnil use.
Instrument	LSR Fortessa (BD Biosciences)
Software	FlowJo 8.1. Data visualization with Graphpad Prism 8.3
Cell population abundance	Single cell line was used (so 100%).
Gating strategy	Cell line expressing AR3C was sorted was as indicated in Figure S11

- [x] Tick this box to confirm that a figure exemplifying the gating strategy is provided in the Supplementary Information.
